# ROBOCOP (ROBOtic Care of Poststroke Pain): Study Protocol for a Randomized Trial to Assess Robot-Assisted Functional and Motor Recovery and Impact on Poststroke Pain Development

**DOI:** 10.3389/fneur.2022.813282

**Published:** 2022-02-18

**Authors:** Loris Pignolo, Paolo Tonin, Pierluigi Nicotera, Giacinto Bagetta, Damiana Scuteri

**Affiliations:** ^1^Regional Center for Serious Brain Injuries, S. Anna Institute, Crotone, Italy; ^2^German Center for Neurodegenerative Diseases (DZNE), Bonn, Germany; ^3^Pharmacotechnology Documentation and Transfer Unit, Preclinical and Translational Pharmacology, Department of Pharmacy, Health and Nutritional Sciences, University of Calabria, Rende, Italy

**Keywords:** robot-assisted neurorehabilitation, ARAMIS, stroke recovery, motor rehabilitation, post-stroke pain

## Abstract

**Background:**

Stroke is one of the most frequent causes of death and disability worldwide. It is accompanied by the impaired motor function of the upper extremities in over 69% of patients up to hemiplegia in the following 5 years in 56% of cases. This condition often is characterized by chronic poststroke pain, difficult to manage, further worsening quality of life. Poststroke pain occurs within 3–6 months. Robot-assisted neurorehabilitation using the Automatic Recovery Arm Motility Integrated System (ARAMIS) has proven efficacy in motor function recovery exploiting the movements and the strength of the unaffected arm. The rationale of the ROBOCOP (ROBOtic Care of Poststroke pain) randomized trial is the assessment of the impact of robot-assisted functional and motor recovery on the prevention of poststroke pain.

**Methods:**

A total of 118 patients with hemiplegic arms due to stroke will be enrolled and randomly allocated with a 1:1 ratio to ARAMIS or conventional neurorehabilitation group. After a baseline screening at hospital discharge, ARAMIS or conventional rehabilitation will be performed for 8 weeks. The primary endpoint is the prevention of the development of poststroke pain and the secondary endpoints are prevention of spasticity and efficacy in clinical motor rehabilitation. The primary outcome measures consist in the visual analog scale and the doleur neuropatique 4 and the secondary outcome measures include: the Modified Ashworth Scale, the Resistance to Passive movement Scale; the Upper Extremity Subscale of the Fugl–Meyer Motor Assessment; the Action Research Arm Test; the Barthel Index for activities of daily living; and the magnetic resonance imaging (MRI) recovery-related parameters. After baseline, both primary and secondary outcome measures will be performed in the following time points: 1 month after stroke (*t*_1_, half of the rehabilitation); 2 months after stroke (*t*_2_, after rehabilitation); and 3 months (*t*_3_) and 6 months (*t*_4_) after stroke, critical for poststroke pain development.

**Discussion:**

This is the first clinical trial investigating the efficacy of robot-assisted neurorehabilitation using ARAMIS on poststroke pain prevention. This study could remarkably improve the quality of life of stroke survivors.

## Introduction

### Background and Rationale

Stroke represents one of the most serious causes of death and disability-adjusted life-years lost (1) destined to keep increasing due to global aging. One of the most common long-term disabilities occurring in over 69% of cerebrovascular lesions consists of impaired motor function of the upper extremities up to hemiplegia in the following 5 years in 56% of cases (2, 3). Stroke is complicated by the occurrence of poststroke pain usually within 6 months from the acute event (4), with poststroke pain syndromes reported in up to 30%−40% of stroke outliving patients (5), chronic in 11–55% of patients (6). The latter include pain caused by different pathophysiology in which nociceptive and neuropathic features can coexist ranging from pain secondary to spasticity, shoulder pain, complex regional pain syndrome (CRPS), and headache, in 1 out of 10 stroke sufferers, that is, migraine-like in 31.3% cases (7), to central poststroke pain (8). Shoulder pain is the most frequent affecting some 30–40% of stroke survivors (6). Lenticulocapsular strokes can cause hemiparesis with consequent shoulder pain (9). The development of poststroke pain correlates to the severity of stroke and paresis is associated with a 3.1-fold higher risk for stroke-related pain (10). Unfortunately, often patients suffering from pain after stroke do not find relief in current analgesic treatment because it is not supported by clinical trials, as a consequence, guidelines are lacking (9). In fact, the systematic review and meta-analysis of literature has highlighted that very few clinical trials assess the efficacy of opioids, the most potent analgesics on poststroke pain, the number of patients is very small and these studies have not been designed specifically for this condition, thus usually not evaluating the correlation between rehabilitation, pain, and physical functioning after stroke (11). Actually, stroke survivors do not even benefit from clinical trials on the novel preventative treatment of migraine, e.g., the anticalcitonin gene-related peptide monoclonal antibody eptinezumab that can provide rapid and longer-lasting action (12). This is often due to the lack of use of pain scales for an objective pain assessment validated in specific populations, e.g., patients affected by stroke, and this problem becomes even more worrying for patients with aphasia and noncommunicative and in the pandemic emergency (13–16). Robot-assisted neurorehabilitation for patients with stroke having the Integrated Robotic System for Stroke (IRSS) prototype Automatic Recovery Arm Motility Integrated System (ARAMIS) has provided a significant improvement of motor recovery (17). It consists of two computer-controlled, symmetric, and interacting exoskeletons that impress to the paretic arm the same strength and movement of the healthy arm and it has been validated in stroke survivors. The rationale for the use of ARAMIS in poststroke neurorehabilitation of the paretic upper limb relies on the possible prevention of aberrant early plasticity. According to our working hypothesis, ARAMIS might improve plasticity in agreement with the concept underlying the Reinforcement-Induced Movement Therapy (18), avoiding the occurrence of spasticity and hypotonia.

### Objectives

In agreement with the Initiative on Methods, Measurement, and Pain Assessment in Clinical Trials (IMMPACT) recommendations that support the importance of physical functioning as the core outcome for pain (19) this randomized controlled clinical trial (Calabria Region Ethics Committee protocol N.244 of 09/21/2021) will be the pilot forming the rational basis for the assessment of the efficacy in the use of ARAMIS to prevent chronic poststroke pain development. In fact, according to our working hypothesis, proprioceptive inputs with high-intensive bilateral movement training of the hemiplegic arm can prevent chronic poststroke pain development within the 3–6 months following stroke.

### Trial Design

This randomized single-center trial will recruit subacute hemiplegic patients of any age with hemiparesis of the arm after stroke. The trial is designed as a prospective, exploratory, and interventional study without drugs. The study does not request the use of drugs. This study protocol follows the Standard Protocol Items: Recommendations for Interventional Trials (SPIRIT) Checklist (20).

## Methods

This trial has been approved by the Calabria Region Ethics Committee (protocol N.244 of 09/21/2021) and the ClinicalTrials.gov ID has been requested. According to the D.lgs 196/2003, the Helsinki agreements and subsequent amendments, the Good Clinical Practice and current legislation, the Guidelines for the treatment of personal data in clinical trials of 24 July 2008, and in accordance with European data protection legislation, each participant or his/her legal representative will be required to sign a consent form as acceptance of all aspects of the study contained in the patient information sheet and as a consequent expression of his willingness to participate in the study. The information sheet will be duly illustrated to the subjects or legal representatives by the study staff and the same staff will ensure that the consent form is properly signed and dated by all the parties involved before any procedure foreseen by the protocol is carried out.

### Study Setting

The study will be carried out at the Sant' Anna Institute of Crotone (Calabria, Italy), which is specialized in motor and cognitive treatment and rehabilitation of neurological diseases.

### Eligibility Criteria

Consecutive patients, admitted to Sant' Anna Institute with the diagnosis of stroke after hospital discharge will be enrolled, accordingly with the following inclusion criteria:

- First-ever stroke within 24–72 h from diagnosis confirmed through functional MRI scan, regardless of the side, location, and extension of the lesion;
-Stroke-related hemiplegic patients of any age at hospital discharge within 48–72 h after stroke with fMRI scan of the area affected;
-Severe upper limb impairment, according to the Upper Extremity Subscale of the Fugl–Meyer Motor Assessment [FMA-UE (0-35) (21)];
-Absence of hemiplegic upper limb-related baseline pain based on the visual analog scale (VAS), the bedside active examination with a numerical rating and, also, the douleur neuropathique en 4 questions (DN4) for the evaluation of neuropathic prestroke pain.

Exclusion criteria will be:

-Bilateral impairment;
-Presence of aphasia;
-Presence of cognitive impairment, as assessed using the cognitive assessment scale for patients with stroke (22), to avoid interference of aphasia or hemispatial neglect that may occur with the commonly used Mini-Mental State Examination (23) and Montreal Cognitive Assessment (24);
-Stroke diagnosis without the occurrence of hemiparesis of the upper limb;
-Previous rehabilitation.

Due to the lack of specific observational tools for poststroke pain in noncommunicative patients, this pilot study will not include patients affected by aphasia and cognitive impairment. The informed consent will be obtained by healthcare operators and patients will be informed about the study and they will be provided with a consent form. No additional consent provisions for biological specimens will be needed since the latter will not be collected.

### Description of the Intervention

**Figure 1:**
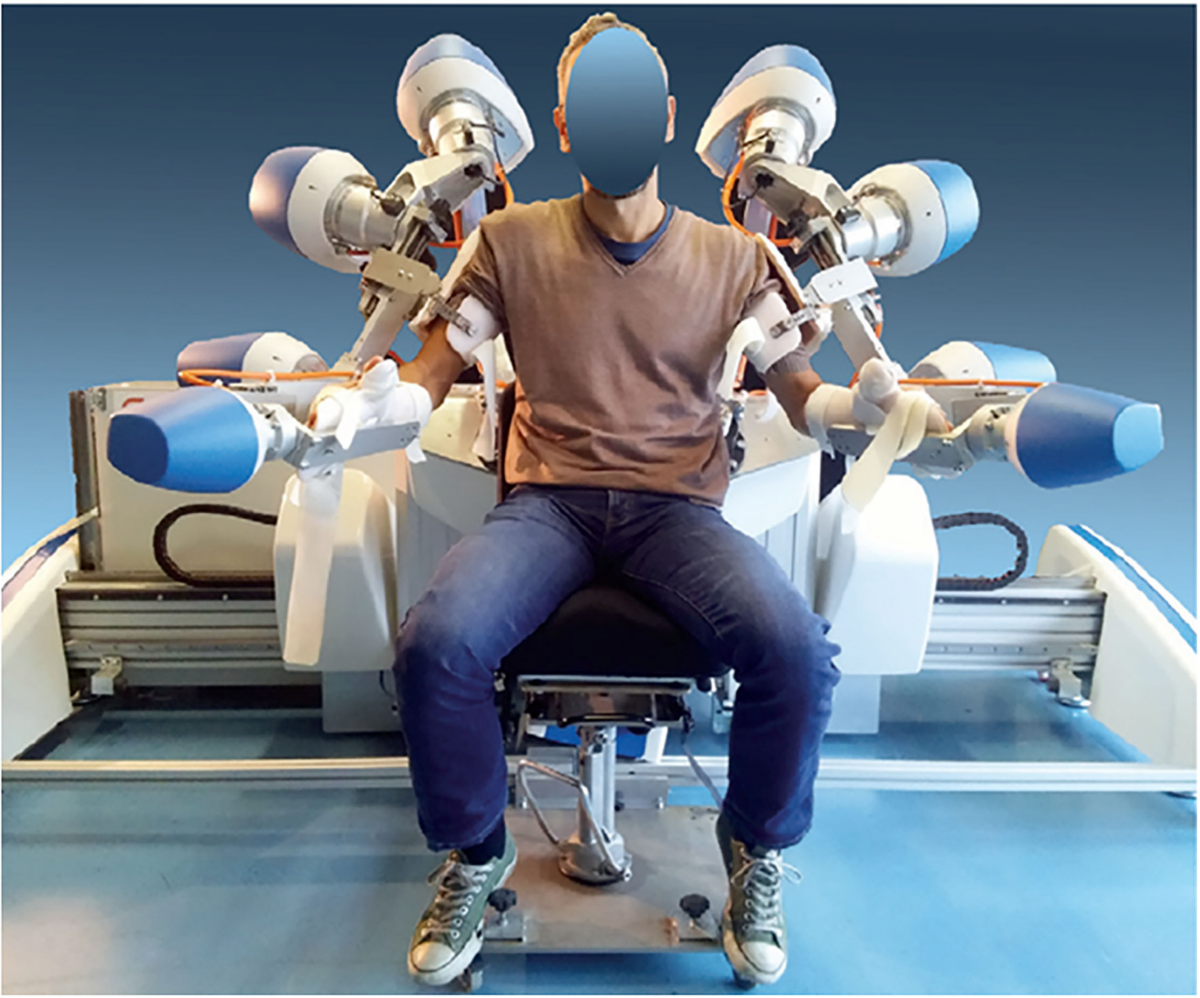
Integrated Robotic System for Stroke (IRSS) prototype Automatic Recovery Arm Motility Integrated System (ARAMIS) [reproduced with permission from Pignolo et al. (25)].

ARAMIS is composed of two computer-controlled, symmetric, and interacting exoskeletons able to compensate for the inadequate strength and accuracy of residual motor function of the paretic arm movements (25) (Figure 1). Through motion capture of the movements of the unaffected arm, the patient can replicate the movements of the healthy arm with the paretic arm in a synchronous, asynchronous, or active-assisted manner (25). Patients in the intervention group will receive only robot-aided and not conventional rehabilitation for a 60 min-session. The robot-assisted neurorehabilitation using ARAMIS and the conventional rehabilitation consists of 60-min sessions every day for 8 weeks (17, 25). In particular, in the first section of neurorehabilitation (1–4 weeks), all the subjects will be subjected to perform a series of asynchronous exercises according to which the paretic limb repeats each exercise 20 times for a total of 200 repetitions per session. During synchronous exercises, the exoskeleton hosting the paretic limb allows the replication of the sample movements of the parallel exoskeleton hosting the unaffected arm in real time. In the asynchronous exercises, the sample movements have been generated previously by the unaffected arm of the patient or by the arm of the therapist. The basic exercises consist in forearm pronation-supination, elbow flexion-extension, shoulder elevation (30°, 60°, and 90°) and abduction-adduction (30°, 60°, and 80°), and circle movement on the frontal axis of the shoulder and its flexion-extension. On the other side, the functional exercise includes: shoulder elevation 90° + forearm pronation-supination and elbow flexion-extension and the contrary (elbow flexion-extension + forearm pronation-supination); shoulder elevation 90° + two elbow intermediate flexion-extension + forearm intermediate pronation-supination. In the following section (5–8 weeks), the asynchronous exercises are reduced to 100 per session and the number of synchronous exercises is increased to keep constant the total number (200/session). The control group will receive conventional rehabilitation. The latter consists of passive mobilization, neuromotor facilitation of shoulder, arm, forearm, and hand muscles and adoption of postures inhibiting pathological synergies in the first 4 weeks. In the following 4 weeks apart from a passive mobilization, neuromotor facilitation, and posture adopting, also the following exercises will be performed: coordination proximal-distal and of ocular-cephalic movements; “reach to indicate”; hand preconfiguration; “reach, touch, and manipulate”; grasping; exercises for manipulative skills, for the adaptation of the hand to the object without visual feedback and the sensitivity, together with biofeedback and electrostimulation cardiovascular conditioning in the sitting posture, conditioning in the upright posture and exercises for the trunk control.

In case of discontinuation of the treatment, the patient will be excluded from the study. The treatment will be performed by healthcare operators preventing lack of adherence.

### Outcomes

The effect of robot-assisted rehabilitation using ARAMIS on the prevention of the development of poststroke pain is the primary endpoint of the ROBOCOP study. The primary outcome measures consist in the assessment of pain through the VAS, including a bedside active examination and probing of somatosensory functions to test touch, temperature, and pain sensations (26). The development of neuropathic components of poststroke pain will be assessed using the DN4. The secondary endpoints include spasticity, tightly linked to pain and developed by up to 40% of patients with hemiparetic stroke, and clinical motor rehabilitation. Spasticity will be assessed using the Modified Ashworth Scale (MAS) (27) and the Resistance to Passive movement Scale (REPAS), based on the Ashworth scale (28), for major joint movements in upper and lower limbs. Clinical motor rehabilitation will be measured according to the Standardizing Measurement in Arm Rehabilitation Trials [SMART] toolbox of the recommended core set of outcome measures for rehabilitation after stroke (29, 30). The latter includes FMA-UE, the Action Research Arm Test, and the activities of daily living/stroke-specific outcomes of the Barthel Index. The clinical motor rehabilitation will also be characterized through structural and functional MRI scan. In particular, structural MRI is intended to evaluate infarct and injury volume (31) and functional MRI to assess the activation volume in the stroke ipsilateral hemisphere during movement of paretic limb, which is related to the extent of behavioral recovery (32). The connection strength between the ipsilesional primary motor cortex and the contralateral regions will be examined (33).

### Participant Timeline

Patients will be randomly allocated to the intervention or control group in a 1:1 allocation ratio. The enrollment will last up to the achievement of the sample size. The most important time points for the assessment of pain development are 3 and 6 months poststroke, with the addition of measurement time points in the subacute phase (29, 34). In fact, differences in motor activity of paretic limbs 24–36 h after symptoms onset, and also at 3 and 6 months (35, 36) have been demonstrated. Therefore, the assessment of the primary and secondary outcomes will be conducted at the following time points: baseline measurement at Sant'Anna Institute admission (24–72 h since stroke event) (*t*_0_); 1 month after stroke (*t*_1_), i.e., at half of the rehabilitation period; 2 months after stroke (*t*_2_), i.e., after the completion of neurorehabilitation with ARAMIS in the intervention group and of conventional rehabilitation in the nonintervention group; 3 months (*t*_3_) and 6 months (*t*_4_) after stroke, since the latter are critical for rehabilitation and pain development. Routine clinical and neurophysiological assessments will be performed before and at the end of the treatment. Loss of tolerance to the procedure up to discontinuation will be recorded on a specific form. The schedule of enrollment, interventions, and assessments is reported in Table 1.

**Table 1:** Schedule of enrollment, interventions and assessments according to Standard Protocol Items: Recommendations for Interventional Trials (SPIRIT).

	**Study period**					
	**Enrolment**	**Allocation**	**Post-allocation**			
**Timepoint**	***–t_**1**_***	**0**	***t_**1**_***	***t_**2**_***	***t_**3**_***	***t_**4**_***
**Enrolment**						
Eligibility screen	X					
Informed consent	X					
Physical examination	X					
Allocation		X				
**Interventions**						
*ARAMIS*			X	X		
Conventional neurorehabilitation			X	X		
**Assessments**						
Primary endpoint variables		X	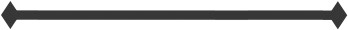			
Secondary endpoint variables		X	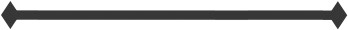			
Assessment of activation volume			X	X		
FAI		X				X

### Sample Size

The calculation of the sample power is based on literature data (25, 36–40) to obtain 25% of improvement in the primary endpoint using G^*^Power 3.1.9.7 (41). The formula for calculating the sample size is given, for two arms of the same size, as it follows:


$$\begin{array}{l} n=\left[{\left(Z_{\alpha /2}+Z_{\beta }\right)}^{2}\times \left(2{\left(\sigma \right)}^{2}\right)\right]/{\left(\mu 1-\mu 2\right)}^{2} \end{array}$$

where,

*n* = sample size required in each group;

*Z*_α/2_ = 1.96, according to 5% level of significance;

*Z*_β_ = 0.84 for 80% power;

σ = standard deviation;

μ1 – μ2 = primary outcome measures significant difference = 6.34 with ARAMIS and 5.4 with conventional rehabilitation (25);

*d* = 0.3.

Therefore, considering a 95% confidence level and a study power of 80% (42), the result will be *n* = 53 per arm.

Adjusted sample size *n*_1_ to account for potential drop-out rates is *n*_1_ = *n*/(1–*d*), where *d* = 10% (43), thus *n*_1_ = 118 patients will be recruited, 59 per each arm.

### Recruitment, Allocation, Concealment, and Blinding

The recruitment will occur at the study setting (Sant' Anna Institute) to reach the target sample size. Patients will be randomly allocated to in a 1:1 allocation ratio to ARAMIS or conventional neurorehabilitation group. Different researchers/operators will independently recruit patients, generate the allocation sequence, and assign participants to interventions and no member of the trial will have access to the codes up to the end. Both males and females will be enrolled due to differences in baseline pain perception (44) and to sex-treatment interactions (45). Therefore, no blocking will occur but minimization will be performed for sex-difference in pain perception, handled to ensure that any individual who might introduce bias is not involved (46). The codes of allocation will be generated using Random Number selection Microsoft Office Excel 2010 (Microsoft, Milan, Italy). Blinding of data managers and biostatisticians after assignment to interventions and also about the nature of the interventions will be ensured (47). Accordingly, who will be in charge of preparing the randomization list will be someone other than the data managers and biostatisticians (47). The allocation will never be revealed up to the end of the trial. Double data entry will be performed by two independent operators for security and data quality. To guarantee the protection of confidentiality, only the responsible secretariat of the clinical center will collect and maintain the personal information of patients. Not having a trial sponsor, auditing will be independent.

### Statistical Analysis

The statistical analysis plan follows the Consolidated Standards of Reporting Trials (CONSORT) evidence-based reporting guideline to ensure research transparency, specifically to trials of nonpharmacologic treatments (NPTs), as rehabilitation (48). The primary (pain) and secondary (spasticity and rehabilitation parameters) outcome measures, collected at prespecified time point *t*_3_ and *t*_4_ vs. *t*_0_, will be expressed as mean and standard error and assessed for statistically significant differences using independent *t*-test for data normally distributed or Mann–Whitney U test for skewed data. Based on the CONSORT NPT item 12b, the evaluation of the outcome measures over time will be carried out through a linear mixed model. The correlation between pain (VAS measure) and recovery (FMA-UE), assessed at *t*_0_, *t*_1_, *t*_2_, *t*_3_, and *t*_4_, will be performed through the Pearson product-moment correlation coefficient (*r*). In agreement with the CONSORT NPT item 12a, the clustering of data will be performed by blinded care providers of the center. Statistical analysis will be performed using SPSS statistics software (Chicago, IL, USA). Values of *p* < 0.05 will be considered statistically significant.

## Discussion

Cerebrovascular accidents are often accompanied by motor disability and chronic pain. Poststroke pain consists of complex syndromes including both nociceptive musculoskeletal and neuropathic features (49). Due to its characteristics, poststroke pain is often underdiagnosed and unrelieved (50). The best management option for chronic pain consists of its prevention. To this aim, this is the first clinical trial to study the use of an IRSS in subacute neurorehabilitation after stroke. In particular, ARAMIS has provided more efficacy than conventional poststroke neurorehabilitation when applied for 7–8 weeks in the improvement of motor function and activities of daily living (17, 25, 51, 52). When the lesion is high after lateral medullary infarction, known as Wallenberg's syndrome, or in the ventroposterior part of the thalamus, central poststroke pain ensues (6). In the event hemiparesis occurs, the healthy arm may offset the effect of spasticity and locomotion (52, 53). Within this frame, ARAMIS may prevent central pain, and, concurrently, may drive correct re-organization in hemiplegic patients capturing the motions and the strength of the unaffected upper limb. In fact, ARAMIS consists of two exoskeletons with 6 degrees of freedom driven by two engines and controlling the shoulder joints to compensate for the inadequate motility and strength of the paretic arm (51). Robot-assisted motor rehabilitation and myoelectrical stimulation are demonstrated to provide reduction of the upper limb impairment (38, 39, 54–56) and pain improvement (40, 57, 58). Also, actigraphic measure systems can predict acute phase stroke prognosis (59), supporting the role of technological progress in stroke. Furthermore, the use of these devices can help the familiarization of poststroke survivors with the latter technology (60). However, a decrease in upper limb impairment does not always correlate to practical and daily functional improvement and this translational aspect needs to be studied for effective neurorehabilitation (61). In fact, the wide multicenter trial for the evaluation of robot-assisted training for the upper limb after stroke (RATULS) has demonstrated that robotic neurorehabilitation does not significantly improve the upper limb function (62). Pain intensity has been positively correlated with time since stroke and negatively with motor function (44), supporting the need to examine the role of robot-assisted rehabilitation in pain processing and to clear the mechanisms necessary for effective functional gain. The latter is a top ten priority for stroke survivors (63). Therefore, the role of ARAMIS-assisted neurorehabilitation in the prevention of poststroke pain occurring 3–6 months after stroke deserves investigation. The efficacy of robot-assisted neurorehabilitation in the prevention of chronic poststroke pain could pose the basis for a remarkable improvement of stroke sufferers' quality of life. Moreover, a better and deeper understanding of robot-related motor learning is needed (55) and, although the stage is a predictor for better rehabilitation outcomes, IRSS neurorehabilitation, without significant differences among the several devices existing (64), has proven promising results also in chronic stroke survivors (65). In this already fragile population, robot-assisted rehabilitation may also reduce the use of analgesics known to be endowed with serious side effects. In fact, fragile populations are often subjected to limited and inappropriate pain treatment (66–68). The next step will consist in investigating the effect of this neurorehabilitation procedure on the use of analgesics, both synthetic and of natural origin, as the essential oil of bergamot endowed with analgesic (69) and flumazenil insensitive anxiolytic-like (70) properties and which has been engineered allowing double-blind clinical trials (71, 72).

## Author Contributions

All authors listed have made a substantial, direct, and intellectual contribution to the work and approved it for publication.

## Funding

DS is a researcher in the frame of the project supported by the Italian Ministry of Health: NET-2016-02361805 (WP 5).

## Conflict of Interest

The authors declare that the research was conducted in the absence of any commercial or financial relationships that could be construed as a potential conflict of interest.

## Publisher's Note

All claims expressed in this article are solely those of the authors and do not necessarily represent those of their affiliated organizations, or those of the publisher, the editors and the reviewers. Any product that may be evaluated in this article, or claim that may be made by its manufacturer, is not guaranteed or endorsed by the publisher.
